# Globozoospermia and lack of acrosome formation in GM130-deficient mice

**DOI:** 10.1038/cddis.2016.414

**Published:** 2017-01-05

**Authors:** Feng Han, Chunyi Liu, Lianjun Zhang, Min Chen, Yang Zhou, Yan Qin, Yaqing Wang, Min Chen, Shuguang Duo, Xiuhong Cui, Shilai Bao, Fei Gao

**Affiliations:** 1 State Key Laboratory of Stem Cell and Reproductive Biology, Institute of Zoology, Chinese Academy of Sciences, Beijing 100101, China; 2 University of Chinese Academy of Sciences, Beijing, China; 3 State Key Laboratory of Molecular and Developmental Biology, Institute of Genetics and Developmental Biology, Chinese Academy of Sciences, Beijing, China

## Abstract

Globozoospermia is a common reproductive disorder that causes male infertility in humans, and the malformation or loss of acrosomes is the prominent feature of this disease. Although the acrosome is thought to be derived from the Golgi apparatus, the detailed molecular mechanisms remain unclear. GM130 is a *cis*-side localized Golgi matrix protein,whereas the physiological functions of this protein remain elusive. Here we showed that inactivation of GM130-caused male infertility in mouse model. The primary defects were the absence of acrosomes, round sperm heads, and aberrant assembly of the mitochondrial sheath, which comprise the characteristic features of human globozoospermia. Further investigation indicated that loss of GM130 did not affect the secretion of pro-acrosomic vesicles, whereas the vesicles failed to fuse into a single large acrosome vesicle. Co-localization of the adaptor protein complex AP1 and *trans*-Golgi network (TGN) protein TGN46 was disrupted, suggesting that the malformation of acrosomes is most likely due to the defect in the sorting and coating of Golgi-derived pro-acrosomic vesicles. Thus, the GM130-deficient mouse provides a valuable model for investigating the etiology of human globozoospermia.

Spermiogenesis is a fundamental process required for the generation of the mature male gamete with a vesicle-like acrosome, a propelling flagellum, and condensed nucleus, which are all necessary for successful fertilization. The acrosome is a cap-like membrane structure that covers the anterior portion of the sperm head. The formation of the acrosome occurs during early stages of spermatid development. The Golgi-derived vesicles accumulate in the concave region of the sperm nucleus, and a single large acrosomic granule is formed by the fusion of small vesicles and attaches to the nuclear envelope by interacting with the acroplaxome.^1^ The size of the acrosome subsequently increases by fusing with more Golgi-derived vesicles and spreading over the anterior nuclear pole. The morphogenic changes in the acrosome during spermiogenesis have been well documented; however, the regulatory mechanisms remain elusive.

Globozoospermia (also referred to as round-headed spermatozoa) is one of the common human reproductive disorders.^2, 3, 4^ The most prominent feature of globozoospermia is that the nucleus of the sperm exhibits a round shape, and the acrosome is malformed or completely absent in severe cases. Globozoospermia is also characterized by an abnormal arrangement of the mitochondria of the spermatozoon.^5^ In mouse models, several genes have been demonstrated to be involved in acrosome biogenesis, and the inactivation of these genes triggers globozoospermia. Pick1 (ref. 6), Gopc,^7^ Vps54 (refs 8, 9) and Hrb^9^ are required for acrosome formation by controlling Golgi vesicle fusion. Zpbp1 (ref. 10), Ck2 (ref. 11), Hsp90b1 (ref. 12) and Gba2 (ref. 13) have more diverse cellular localizations and functions. Our recent study has demonstrated that the autophagy process is also required for acrosome biogenesis, and the inactivation of the autophagy-associated gene Atg7 causes malformation of the acrosome and a round-headed spermatid.^14^

GM130 was first isolated from the Golgi matrix as a structural protein which is localized at the *cis*-side of the Golgi complex.^15, 16^ As a member of the golgin family, the function of GM130 has been investigated using *in vitro* systems.^16, 17^ Together with p115, giantin, and GRASP65, GM130 is thought to play roles in ER-derived vesicle tethering and fusion at the Golgi membrane, thereby maintaining the Golgi structure integrity.^18, 19, 20, 21^ Emerging evidence has indicated that GM130 also has essential roles in the control of cell polarity, cell division, and cell migration.^22, 23, 24, 25^ A recent study has demonstrated that GM130 is involved in the Golgi-derived spindle assembly via TPX2 activation and microtubule capture.^26^ However, as a Golgi matrix protein, the physiological functions of GM130 in acrosome biogenesis have not been investigated. To determine the functions of the Golgi apparatus in acrosome formation during spermatogenesis, a GM130 gene knockout mouse model was generated. We found that the sperm from the *GM130*^−/−^mice were round-headed, with complete absence of acrosome and mitochondrial sheath, and they exhibited characteristics of the sperm present in the human disease globozoospermia. We also demonstrated that the loss of GM130 did not affect the secretion of pro-acrosomic vesicles from the Golgi apparatus, whereas the vesicles failed to move to the concave region of the spermatid nucleus and fuse into a single large acrosomic granule. The loss of GM130 probably causes the defect of sorting and coating of Golgi-derived pro-acrosomic vesicles, which in turn leads to the malformation of the acrosome.

## Results

### Aberrant spermiogenesis was identified in *GM130*^*−/−*^mice

*GM130*^−/−^mice were appeared grossly normal at birth; however, only 50% of the mice survived until the adult stage, and the body size was significantly reduced compared with that of the control littermates. The mRNA level of GM130 in different organs of *GM130*^−/−^mice was verified by Real-time PCR using a primer within the deleted exon (exon 14). As shown in Supplementary Figure S2B, the expression of mRNA from wild-type allele was dramatically reduced in *GM130*^−/−^mice, indicating that GM130 was completely inactivated.

**Figure 1:**
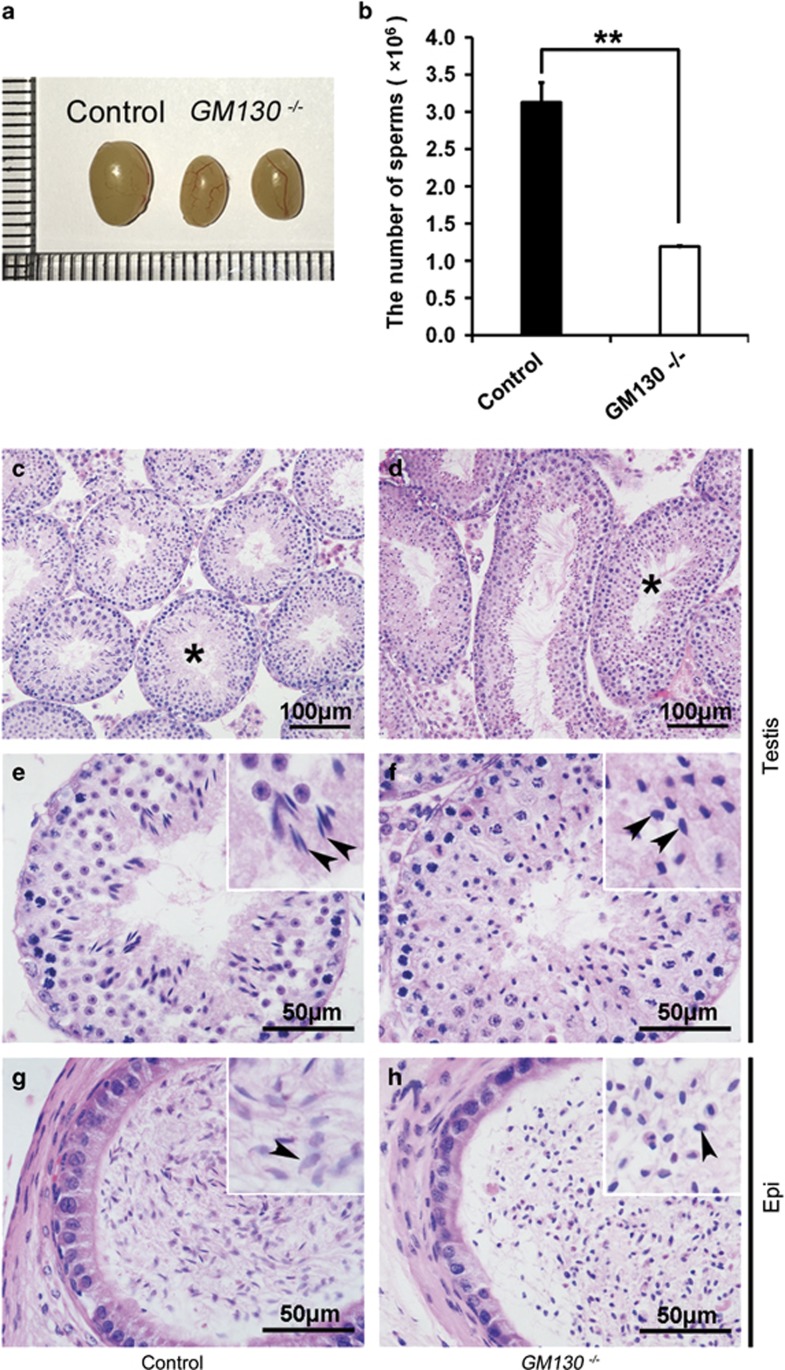
Decreased sperm count and abnormal sperm heads in GM130-deficient mice at 2 months of age. (**a**) The size of the testes from *GM130*^−/−^mice was smaller than that of control mice. (**b**) The sperm number per mouse was reduced ~60% in the GM130-deficient males. The data are represented as the mean±S.E.M. of three independent experiments (*N*=6). The morphology of the seminiferous tubules and sperm from control and *GM130*^−/−^mice was examined by H&E staining. The seminiferous tubules were grossly normal in *GM130*^−/−^mice (**d**) compared with the control mice (**c**). Normal sperm with crescent-shaped heads were observed in both testes (**e**, arrowheads) and epidydimes (**g**, arrowheads) of control mice. The sperm heads in both testes (**f**, arrowheads) and epidydimes (**h**, arrowheads) of GM130-deficient mice were in round-shaped

**Figure 2:**
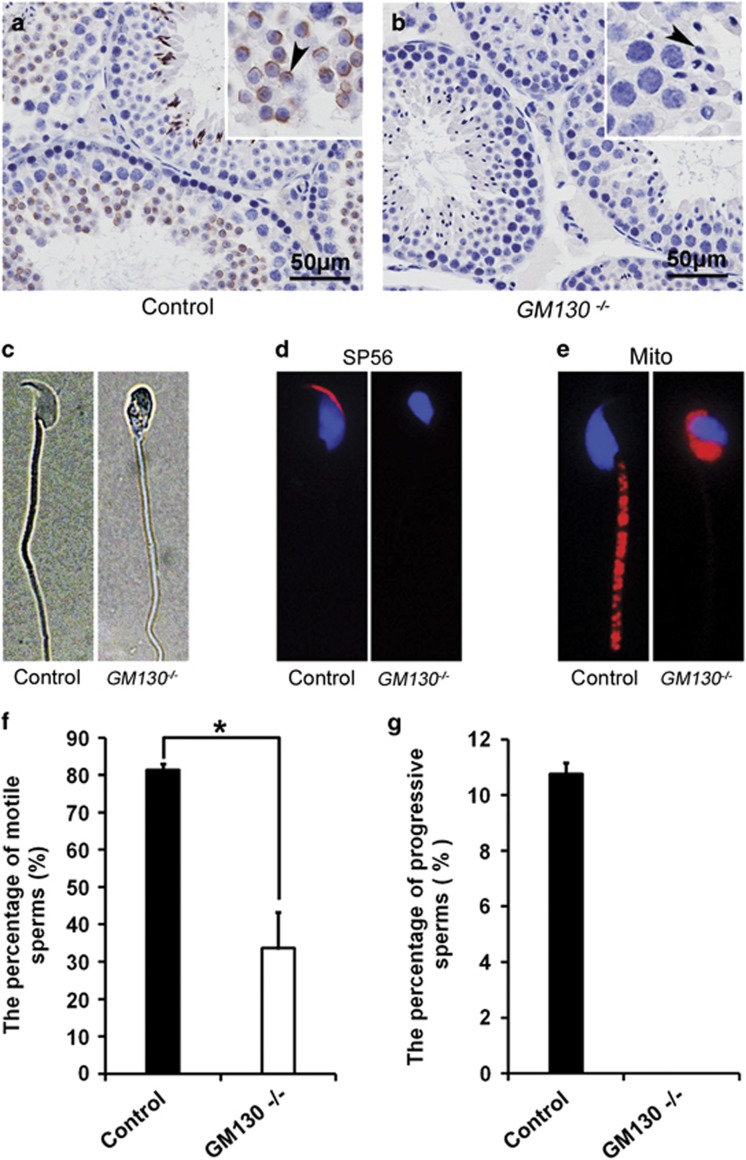
Defects of morphogenesis and motility in GM130-deficient sperm from 2-month-old mice. The morphology of sperm was examined by immunohistochemistry and immunosenesence. Acrosomes were labeled with anti-Afaf antibody in control testes (**a**, arrowheads), and no Afaf signal was detected in GM130-deficient testes (**b**, arrowheads). (**c**) Single sperm image indicated the morphology of control and GM130-deficient sperm. (**d**) Acrosome-specific protein SP56 (red) was detected in the control sperm, but not in the sperm from *GM130*^−/−^mice. (**e**) Mitotracker-positive mitochondrial sheath (red) was observed in the mid-piece of control sperm tails, but not in the tails of GM130-deficient sperm. In contrast, Mitotracker-positive mitochondria (red) were located in the sperm head and surrounded the nucleus. The motility of sperm from control and *GM130*^−/−^mice was analyzed by CASA assay. The percentages of motile (**f**) sperm were substantially reduced in the *GM130*^−/−^mice compared with the control mice (*P<0.05 versus control). No progressive sperm were noted in *GM130*^−/−^testis (**g**)

To investigate the functions of GM130 in spermatogenesis, the morphology and histology of testes from control and *GM130*^−/−^mice were examined by H&E staining. The size of the testes from the *GM130*^−/−^mice was slightly smaller than the control mice (Figure 1a), and the weights of testes in *GM130*^−/−^mice was reduced ~50% (Supplementary Figure S1A and B). However, the ratio of testes weight/body weight was not significantly changed between control and *GM130*^−/−^mice (Supplementary Figure S1C). The histology of the seminiferous tubules was grossly normal (Figure 1d) as compared with the control testes (Figure 1c). Multiple layers of germ cells were identified in the seminiferous tubules of both the control (Figure 1c) and *GM130*^−/−^ (Figure 1d) testes. Mature sperm with crescent-shaped heads were identified in the control testes (Figure 1e) and epididymes (Figure 1g) with higher magnification images. In contrast, all sperm heads in the testes (Figure 1f, inset, black arrowheads) and cauda epididymes from the *GM130*^−/−^ (Figure 1h, inset, black arrowheads) mice were round or ovoid shaped. The defects were clearly indicated by the single sperm image shown in Figure 2c. We then stained the samples for Afaf (also referred to as MN9), an acrosome-specific protein.^27, 28^ The acrosomes at different developmental stages were all labeled with anti-Afaf antibody (Figure 2a) in the control mice, whereas no Afaf signal was detected in the *GM130*^−/−^ (Figure 2b) testes. The single sperm immunostaining results also indicated that the acrosome-specific protein SP56 was completely absent in the GM130-deficient sperm (Figure 2d). These findings indicated that the acrosome was absent in the GM130-deficient sperm. The mitochondrial sheath, which was responsible for sperm movement, also exhibited a severe defect in the GM130-deficient sperm. Mitotracker staining results demonstrated that the mitochondrial sheath was completely absent in the mid-piece of the sperm in the *GM130*^−/−^mice. In contrast, Mitotracker-positive mitochondria were located at the sperm head and surrounded the deformed nucleus (Figure, right panel), thus suggesting that the mitochondrial sheath was not properly assembled in the *GM130*^−/−^mice. The quantification results indicated that the total number of sperm in the cauda epididymes was reduced ~60% in the GM130-deficient mice compared with the control males (Figure 1b). The percentage of motile (Figure 2f) and progressive (Figure 2g) sperm were also substantially reduced in the *GM130*^−/−^mice. These abnormalities were reminiscent of the defects associated with globozoospermia, a human infertility disorder.^29^

### Defects of spermiogenesis were identified in *GM130*^*−/flox*^*; Stra8-Cre* mice, but not *GM130*^*−/flox*^*; AMH-Cre* mice

**Figure 3:**
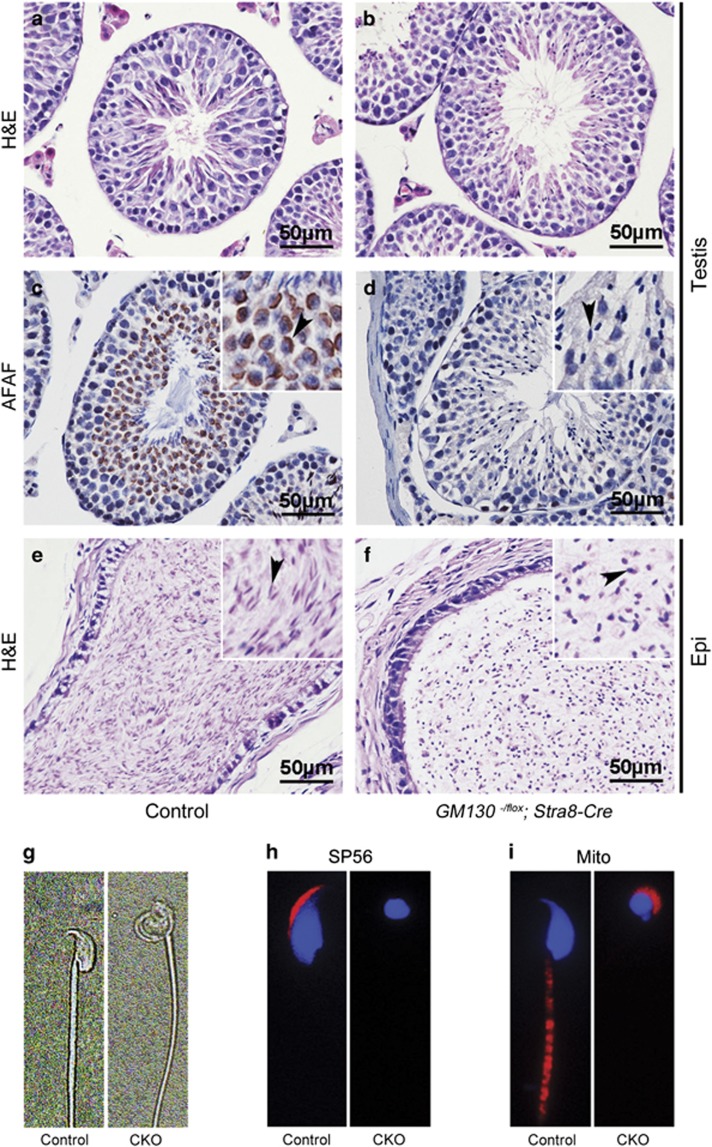
Defect of spermiogenesis was observed in *GM130*^−/flox^*; Stra8-Cre* mice at 2 months of age. The morphology of the seminiferous tubules and sperm was exmained by H&E staining and immunosenesence. The seminiferous tubules were grossly normal in the *GM130*^−/flox^*; Stra8-Cre* mice (**b**) compared with the control mice. (**a**) Acrosomes were labeled with anti-Afaf antibody in control testes (**c**, arrowheads), whereas no Afaf signal was detected in the sperm of the *GM130*^−/flox^*; Stra8-Cre* mice (**d**, arrowheads). Normal sperm with crescent-shaped heads were observed in the epididymides (**e**, arrowheads) of the control mice. The sperm heads in the epididymides (**f**, arrowheads) of the *GM130*^−/flox^*; Stra8-Cre* mice were round. (**g**) single sperm image indicated the morphology of control and *GM130*^−/flox^*; Stra8-Cre* sperm. (**h**) Acrosome-specific protein SP56 was identified in the control sperm, but not in the sperm of the *GM130*^−/flox^*; Stra8-Cre* mice. (**i**) Mitotracker-positive mitochondrial sheath was observed in mid-piece of control sperm, but not in the tails of sperm obtained from the *GM130*^−/flox^*; Stra8-Cre* mice. In contrast, Mitotracker-positive mitochondria were located in the sperm heads and surrounded the nuclei

GM130 is a Golgi-associated protein, which is ubiquitously expressed in all cell types, and the deletion of this gene also causes other defects, including growth retardation (unpublished data). To exclude the possibility that the aberrant spermiogenesis is a consequence of developmental defects of other organs following GM130 inactivation, GM130 was specifically inactivated in germ cells by crossing *GM130*^*flox/flox*^ mice with *Stra8-Cre* transgenic mice in which Cre was specifically activated in male germ cells at ~3 days after birth.^30^ *GM130*^−/flox^*; Stra8-Cre* mice were obtained at the normal Mendelian ratio, and no overt abnormalities were observed. The morphology of the seminiferous tubules was grossly normal in the *GM130*^−/flox^*; Stra8-Cre* testes (Figure 3b) compared with the control testes (Figure 3a), whereas no Afaf signal was detected in most of the germ cells of the *GM130*^−/flox^*; Stra8-Cre* mice (Figure 3d, arrowheads). The sperm heads in epididymes (Figure 3f, arrowheads) of the *GM130*^−/flox^*; Stra8-Cre* mice were round, and a single sperm image also demonstrated the malformed sperm heads in the *GM130*^−/flox^*; Stra8-Cre* testes (Figure 3g). In addition, the acrosome-specific protein SP56 was not detected in the sperm from the *GM130*^−/flox^*; Stra8-Cre* mice (Figure 3h), and the Mitotracker-positive mitochondrial sheath was absent in the mid-piece of the sperm (Figure 3i). In contrast, Mitotracker-positive mitochondria were located in the sperm head and surrounded the nucleus (Figure 3i). These defects were similar to the defects identified in the *GM130*^−/−^mice. To further examine the functions of GM130 in Sertoli cells, *GM130*^*flox/flox*^ mice were crossed with *AMH-Cre* transgenic mice. We determined that the spermatogenesis was normal in the *GM130*^−/flox^*; AMH-Cre* male mice. As shown in Supplementary Figure S3, the nucleus of the sperm from the *GM130*^−/flox^*; AMH-Cre* mice was crescent-shaped (B, D, F and G), and the acrosome-specific protein SP56 was detected in both control and *GM130*^−/flox^*; AMH-Cre* sperm (H). The mitochondrial sheath was also well assembled in the *GM130*^−/flox^*; AMH-Cre* sperm (I). These findings suggest that GM130 is involved in spermiogenesis in a cell autonomous manner, and the inactivation of this gene in Sertoli cells does not affect germ cell development.

### GM130 deficiency led to acrosome malformation

**Figure 4:**
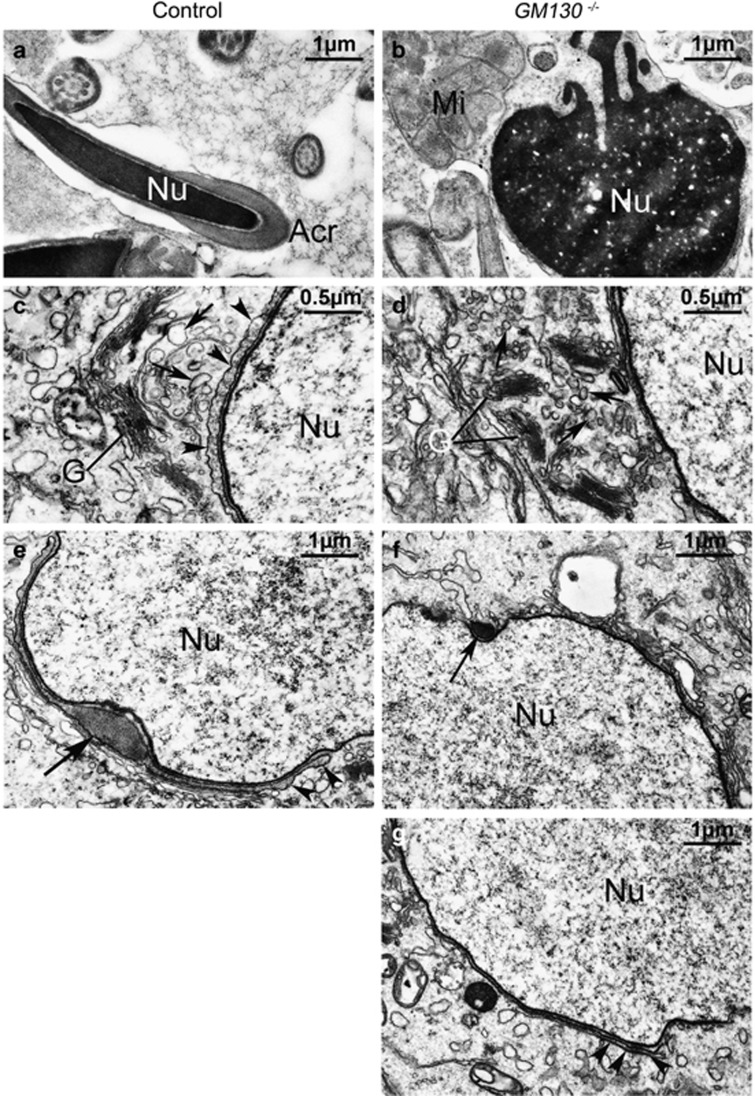
Ultrastructural analysis of spermatogenic cells from control and GM130-deficient mice at 2 months of age. The ultrastructure of sperm was exmianed by TEM. (**a**) In the control mice, the nuclei of the mature sperm was elongated and covered with acrosome. (**b**) The nucleus of the sperm from the GM*130*^−/−^mice did not elongate and remained round in the maturation phase. A cluster of mitochondria was also observed close to the nucleus. A single Golgi apparatus and several large Golgi-derived vesicles (black arrows) were identified in the control spermatids (**c**). The Golgi apparatus was fragmented into several small pieces, and numerous small Golgi-derived vesicles (black arrows) were observed in the GM130-deficient spermatids (**d**). Acrosome granules (**c**, arrowheads and **e**, arrow) were attached to the nuclear envelopes at the Golgi phase in the control sperm. An electron dense acrosome matrix (**f**, arrow) and a thin layer of acrosomal sac (**g**, arrowheads) were also identified in the GM130-deficient sperm. Mi, Mitochondria; Nu, Nucleus; Acr, Acrosome; G, Golgi apparatus

To further investigate the defects of spermatogenesis in *GM130*^−/−^mice, a transmission electron microscope (TEM) experiment was performed. In the epididymes of the control mice, the elongated nucleus of the mature sperm was covered with a cap-like acrosome (Figure 4a). The nucleus of the spermatid in the *GM130*^−/−^mice was round and less condensed. The mitochondria were aggregated and wrapped around the nuclei of the sperm (Figure 4b), which was consistent with the immunostaining results. A single Golgi apparatus and several large Golgi-derived vesicles (Figure 4c, black arrows) were observed in the control spermatids (C). In the *GM130*^−/−^mice, the Golgi apparatus was fragmented into several small pieces, and numerous Golgi-derived small pro-acrosomic vesicles were accumulated in the medulla region (Figure 4d, black arrows). However, these vesicles did not fuse together to yield the large acrosomic vesicle present in the control mice (Figure 4c, black arrowheads). These findings indicate that GM130 is required for the integrity maintenance of the Golgi apparatus; however, it is not required for the secretion of pro-acrosomic vesicles. A single acrosome granule with a dark acrosome matrix was attached to the nuclear envelope in the cap phase of the control sperm (Figure 4e, black arrows). A small acrosome matrix (Figure 4f, black arrows) and a very thin layer of acrosome granule (Figure 4g, black arrowheads) were also present in the sperm of the *GM130*^−/−^mice; however, it did not expand and increase in size. These findings indicate that the initiation of acrosome biogenesis in *GM130*^−/−^mice is not affected; however, additional Golgi-derived pro-acrosomic vesicles cannot be recruited to form a normal acrosome.

### Localization of GM130-interacting proteins was altered in *GM130*^*−/−*^mice

**Figure 5:**
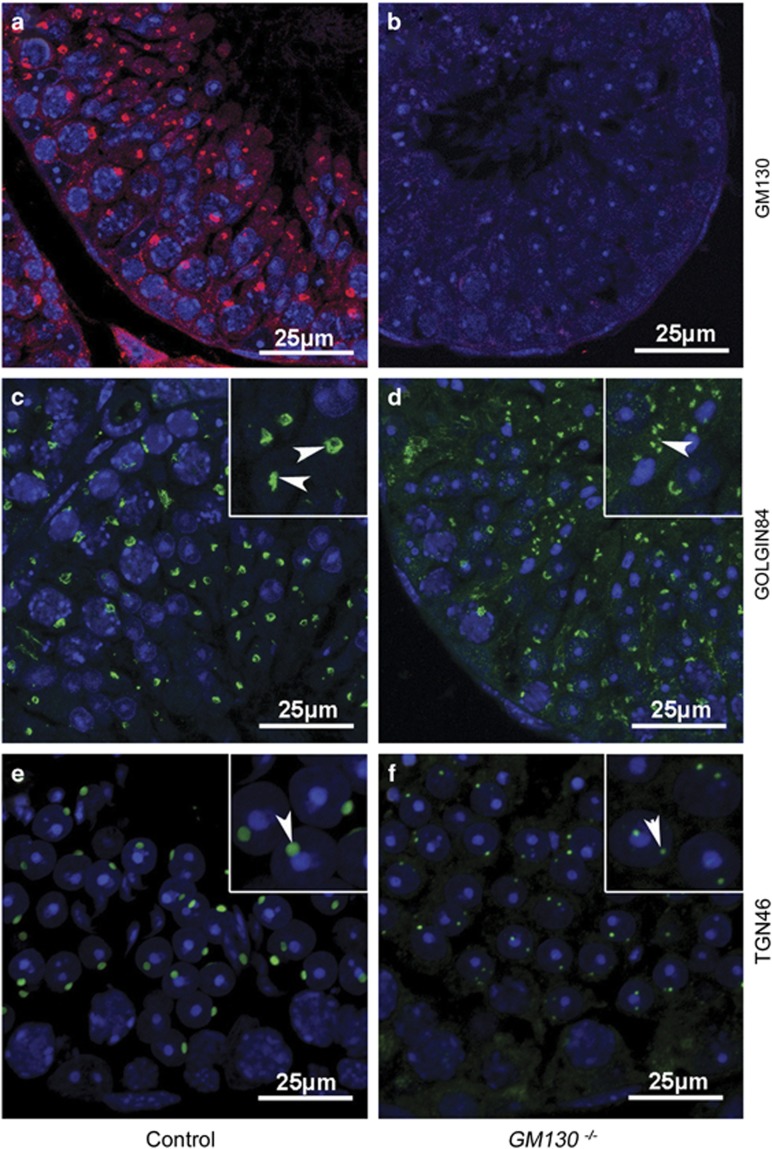
Immunofluorescence of Golgi-specific proteins. The expression of Golgi-specific proteins in both the control and GM130-deficient testes was examined via immunofluorescence. GM130 protein was detected in the germ cells of control testes (**a**, red), but not the *GM130*^−/−^testes (**b**). Golgin84 protein was identified in both control (**c**, white arrowheads) and *GM130*^−/−^testes (**d**, white arrowheads). However, the Golgin84-positive punctae in the *GM130*^−/−^testes were smaller than the control testes. TGN46 protein was also identified in both control (**e**, white arrowhead) and *GM130*^−/−^(**f**, white arrowhead) testes, and the TGN46-positive punctae in the *GM130*^−/−^testes were also smaller than the control testes

To investigate the underlying mechanism that causes the malformation of acrosomes in *GM130*^−/−^mice, the expression of Golgi-associated and GM130-interacting proteins was examined by immunostaining and western blotting. As shown in Figure 5, GM130 protein was detected in the germ cells of the control testes (A, red), whereas no GM130 signal was observed in the *GM130*^−/−^testes (B). These results indicated that the *GM130* gene was completely inactivated in the *GM130*^−/−^mice. Golgin84 (C, D, green) and TGN46 (E, F, green) were detected in the germ cells of both control (C, E) and GM130-deficient (D, F) testes. However, the Golgin84-positive (D, inset, white arrowheads) and TGN46-positive (F, inset, white arrowheads) punctae were substantially smaller than the control mice (C, E, inset, white arrowheads).

**Figure 6:**
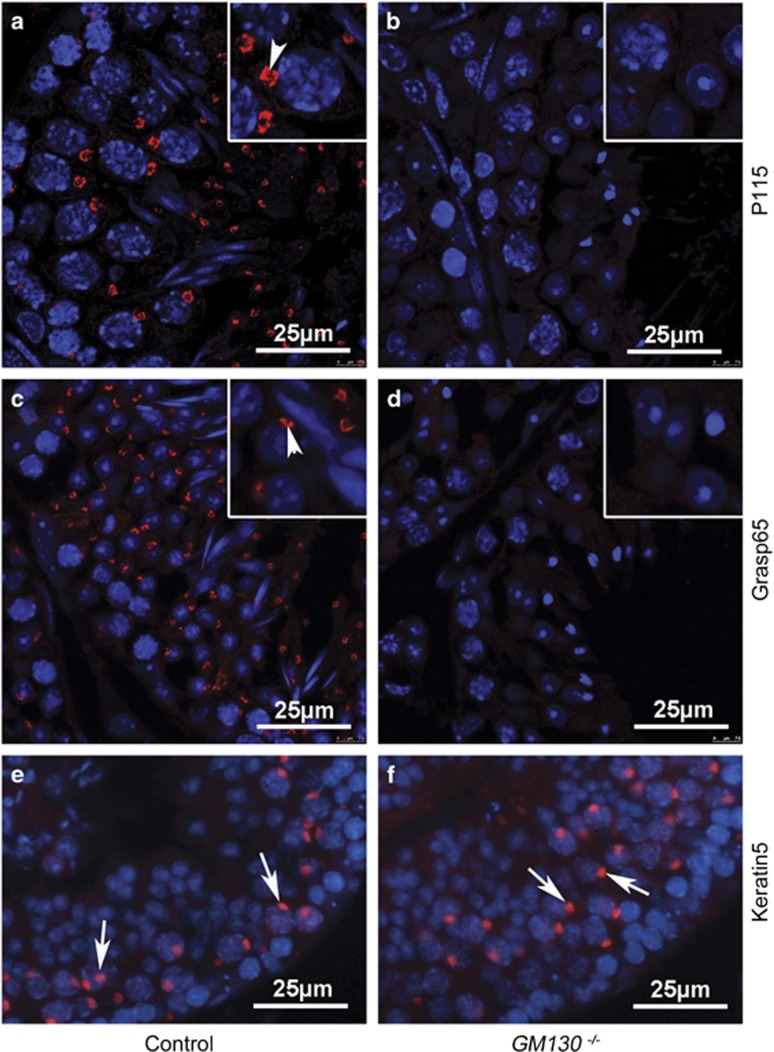
Immunofluorescence of P115, Grasp65 and Keratin5. The expression of GM130-interacting proteins and the acrosome-nucleus interacting protein was examined via immunofluorescence. P115 (**a**, white arrowhead) and Grasp65 (**c**, white arrowhead) proteins were detected in the germ cells of control testes, whereas both P115 (**b**) and Grasp65 (**d**) proteins were absent in the *GM130*^−/−^testes. Keratin5 was expressed in the spermatids of both the control (**e**, white arrows) and *GM130*^−/−^(**f**, white arrows) testes

P115 and GRASP65 are two important GM130-interacting proteins involved in the intercisternal transport in the Golgi stack, as well as transcytosis.^31, 32, 33, 34^ The immunofluorescence results indicated that both P115 (Figure 6a, red) and GRASP65 (Figure 6c, red) proteins were present in the control testes and were completely absent in the GM130-deficient testes (Figures 6b and d). These findings indicated that P115 and GRASP65 proteins could not be recruited to the Golgi stack after GM130 inactivation, which probably resulted in the fragmentation of the Golgi complex. Acroplaxome is a cytoskeletal scaffold that contains F-actin and Keratin 5, which anchors the developing acrosome to the nuclear envelope,^35^ and acroplaxome formation defects also cause the malformation of the acrosome and globozoospermia.^35, 36, 37^ The expression of Keratin 5 was analyzed by immunofluorescence. As shown in Figure 6, Keratin 5 was expressed in the spermatids of both control (Figure 6e, white arrows) and *GM130*^−/−^(Figure 6f, white arrows) testes, and no difference was noted between the control and GM130-deficient germ cells.

### Co-localization of the adaptor protein complex AP1 and TGN46 was disrupted in germ cells of *GM130*^*−/−*^mice

**Figure 7:**
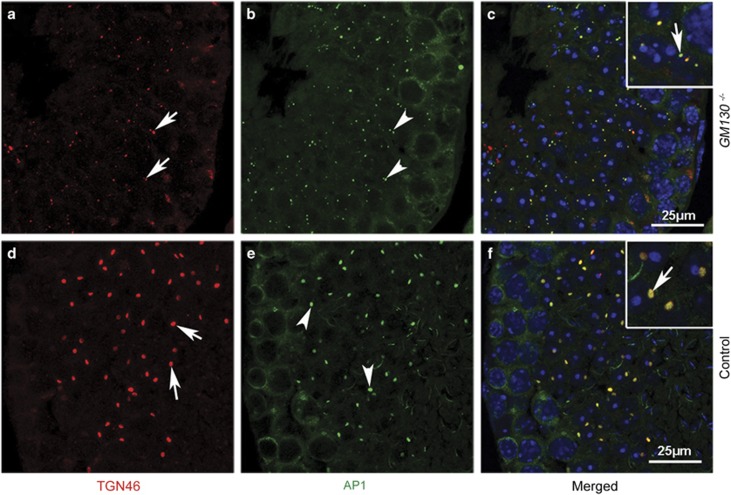
Co-localization of TGN46 and AP1 was disrupted in 2 months GM130-deficient spermatids. The expression of AP1 and TGN46 was examined by immunofluorescence. TGN46 (**d**, red) and AP1 (**e**, green) proteins were detected in the germ cells of control testes, and these two proteins were completely co-localized (**f**, inset, white arrow). TGN46 (**a**, red) and AP1 (**b**, green) proteins were also detected in the GM130-deficient germ cells, whereas the TGN46 and AP1-positive punctae were substantially smaller than those in the control germ cells, and a substantial portion of the AP1-positive punctae were not co-localized with TGN46 (**c**, inset, white arrow)

**Figure 8:**
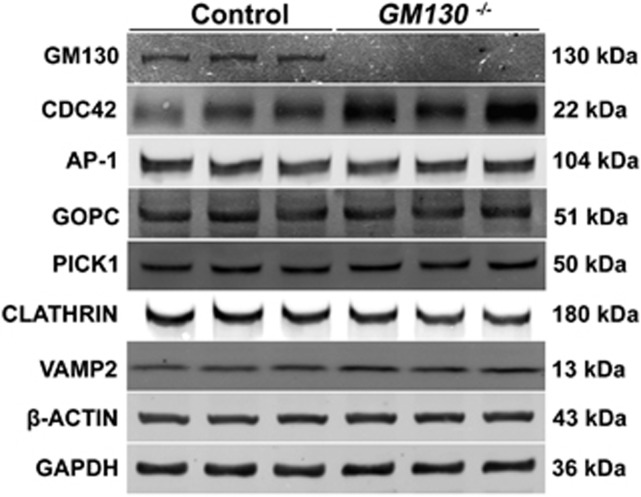
Expression of globozoospermia-related and spermiogenesis-associated proteins. The expression of globozoospermia-related proteins (GOPC and PICK1) was not altered in the *GM130*^−/−^germ cells. The expression of vesicle trafficking-related proteins (AP1, CLATHRIN and VAMP2) was also not altered in the *GM130*^−/−^germ cells. The CDC42 protein level was significantly increased in the GM130-deficient germ cells

It has been reported that the inactivation of Asn-Pro-Phe (NPF) motif-containing protein Hrb also causes the globozoospermia-like phenotype in a mouse model.^9^ In Hrb-deficient germ cells, the interaction between the transport vesicle adaptor protein Eps15 and the AP1 complex is disrupted, thus indicating that the AP1 complex has a critical role in the docking and/or fusion of Golgi-derived pro-acrosomic vesicles. In this study, the localization of the AP1 complex was examined via the immunofluorescence of γ-adaptin. The γ-adaptin protein was prominently present in the small punctae in the medulla (Figure 7e, green), which co-localized with the *trans*-Golgi-specific protein TGN46 (Figure 7f, inset, white arrow) in control germ cells. TGN46 (Figure 7a, red) and AP1 (Figure 7b, red) proteins were also detected in the GM130-deficient germ cells, whereas the TGN46 and AP1-positive punctae were substantially smaller than those in control germ cells, and a large portion of AP1-positive punctae were separated with TGN46-positive punctae (Figure 7c, inset, white arrow). However, the western blot results indicated that the total protein levels of AP1, Clathrin, and another vesicle transport related protein, VAMP, were not changed in GM130-deficient germ cells (Figure 8). The localization of AP1 and GM130 was also examined by immunofluorescence. As shown in Supplementary Figure S4, in the control germ cells, the AP1-positive signal (D, green) was detected in the medulla region, which exhibited a crescent-shaped GM130 signal (F, red). The AP1 and GM130 proteins were adjacent but not co-localized (F, white arrow). The AP1 signal (A, C, green) was also detected in the germ cells of *GM130*^−/−^testes, whereas the AP1-positive punctae were fragmented and smaller than those in the control germ cells (C, white arrows).

### Expression of PICK1 and GOPC was not changed in germ cells of *GM130*^*−/−*^testes

The GOPC protein is localized at the *trans*-Golgi region of round spermatids, and the lack of this protein causes malformation of the acrosome.^7^ Protein interacting with C kinase 1 (PICK1) deficiency also causes male infertility in mice by disrupting acrosome formation.^6^ To determine whether the malformation of the acrosome in GM130-dificient germ cells is a result of the aberrant expression of these two proteins, immunostaining and western blot assays were performed. PICK1 protein was identified in the germ cells of both *GM130*^−/−^(Supplementary Figure S5A and C, red) and control (Supplementary Figure S5D and F, red) testes by immunofluorescence. PICK1 and GM130 were not co-localized in the control germ cells (Supplementary Figure S5F). The western blot results showed that the PICK1 and GOPC protein levels were not changed in the *GM130*^−/−^testes compared with the control testes (Figure 8).

### Cytoskeleton was disorganized in *GM130*^*−/−*^testes

The expression of F-Actin and β-Tubulin was examined by immunofluorescence. In control elongated spermatids, the actin bundles (Supplementary Figure S6A, inset, white arrowheads) were symmetrically localized at both sides of the nucleus. In contrast, the actin bundles were disorganized in the GM130-deficient spermatids, which were detected at only one side of the nucleus (Supplementary Figure S6B, inset, white arrowheads) in most of the spermatids. Microtubules were also assembled symmetrically (Supplementary Figure S6C, inset, white arrowheads) in the elongated spermatids of the control testes. In the GM130-deficient spermatids, the microtubules (Supplementary Figure S6D, inset, white arrowheads) were disorganized and less condensed. Cdc42 is a conserved member of the Rho family of small GTPases, which regulates the assembly of the actin cytoskeleton.^38^ Cdc42 is localized to the Golgi apparatus and is involved in protein transport and the recruitment of regulators of the actin cytoskeleton.^39, 40, 41^ In the present study, we determined that the Cdc42 protein level was significantly increased in the GM130-deficient testes compared with the control testes (Figure 8).

## Discussion

The acrosome is a specialized organelle that covers the anterior portion of the sperm head and is thought to be formed by the fusion of pro-acrosomal vesicles derived from the *trans*-Golgi network.^1, 42^ GOPC (Golgi-associated PDZ- and coiled-coil motif-containing protein) is a Golgi-associated protein, which is localized in the medulla of round spermatids, as well as at the *trans*-Golgi cisternae. A previous study has demonstrated that GOPC-deficient males are infertile with globozoospermia. The primary defect in this mouse model is the fragmentation of acrosomes in early round spermatids, and several discontinued acrosomic vesicles attached to the nucleus envelope have been identified in step 2–3 spermatids.^7^ Further investigation has indicated that the malformation of the acrosome in the GOPC-deficient mice is a result of the aberrant assembly of the peri-nuclear structure in round spermatids, which, in turn, causes the detachment of pseudoacrosomes from the nuclear envelope and disappearance from the peri-nuclear region by spermiation.^43^ Mutation of another Golgi-associated protein, Golga3, also causes male infertility in a mouse model. The defects in the Golga3 mutant male include the blockage of meiosis initiation, and most germ cells die between 15 and 18 days postpartum (dpp).^44^

In this study, we demonstrated that a deficiency of the Golgi matrix protein GM130 in mice causes male infertility. The major defects in the sperm include the absence of acrosomes, round sperm heads, and an aberrant arrangement of mitochondrial sheaths, which represent the prominent features of the human disease globozoospermia.^29^ In the GM130-deficient mouse model, only a small electron dense acrosome matrix and a very thin layer of acrosome sac were observed in the early stage spermatids, and the acrosome structure was completely absent at the later stages. The phenotypes were more severe than that of the GOPC-deficient mouse model.

Previous studies have demonstrated that GM130 has important roles in vesicle tethering and fusion at the Golgi membrane to maintain the integrity of the Golgi structure. In the present study, we also demonstrated that the Golgi apparatus was fragmented into several small pieces in germ cells after GM130 inactivation. This finding further confirmed that GM130 is required for the integrity maintenance of the Golgi structure. A substantial number of small vesicles were observed by TEM in the medulla region between the Golgi apparatus and nucleus in the round spermatids of the *GM130*^−/−^mice, thus indicating that the budding of pro-acrosomal vesicles from the *trans*-side of the Golgi apparatus was probably not affected. However, the size of the Golgi-derived vesicles in the GM130-deficient spermatids was substantially smaller than that of the control germ cells. These findings suggest that the Golgi-derived small vesicles failed to fuse with each other and formed a large granule after GM130 inactivation.

During acrosome formation, proteins are sorted to multiple intracellular and extracellular destinations from the *trans*-Golgi network (TGN). Clathrin is involved in the coating of transport vesicles from the TGN by interacting with the heterotetrametric adaptor protein complex AP1.^45^ In the Hrb knockout mouse model, the interaction between the transport vesicle adaptor protein Eps15 and the AP1 complex is disrupted,^9^ thus indicating that the AP1 complex has a critical role in the docking and/or fusion of Golgi-derived vesicles. In the present study, we demonstrated the co-localization of TGN46 and AP1 was disrupted after GM130 inactivation. These findings suggest that the sorting and coating of secreted vesicles from the *trans*-Golgi is damaged in the absence of the *cis*-side protein GM130. The pro-acrosomic vesicles in the GM130-deficient germ cells are probably not properly assembled, thus causing the failure of vesicle fusion.

This phenotype of Hrb-deficient mice is reminiscent of the phenotype exhibited by GM130-deficient mice. However, the Hrb protein is localized at the outer membrane of the acrosome, whereas GM130 is localized at the *cis*-Golgi apparatus. Thus, the malformation of the acrosome in GM130-deficient mice is probably not related to Hrb. PICK1 protein is highly expressed in round spermatids and localizes to the vesicles between the Golgi apparatus and the acrosomes. The primary defect in the germ cells of Pick1 knockout mice is the fragmentation of acrosomes, which leads to round-headed sperm, a reduced sperm count, and severely impaired sperm motility.^6^ In the GM130-deficient mouse model, the acrosome formation defect was identified earlier than in the PICK1-deficient mice, and the expression of PICK1 protein was also not affected. These findings indicate that the malformation of acrosomes in *GM130*^−/−^mice is not a result of the dysregulation of PICK1 expression.

F-actin-based microfilament and microtubulin-based microtubule also have important roles in spermiogenesis. The transient microtubule-containing structure manchette and the peri-nuclear ring adjacent to the marginal ring of the acroplaxome are both involved in shaping the heads of spermatids, transporting vesicles and macromolecules to the centrosome and developing spermatid heads. In this study, we demonstrated that the microfilament and microtubule were both disorganized in the GM130-deficient germ cells. Cdc42 is a member of the Rho family of small GTPases, which are established regulators of the actin cytoskeleton.^38^ A previous study has demonstrated that the GM130-RasGRF complex is a regulator of Cdc42 at the Golgi, and silencing of GM130 results in RasGRF-dependent inhibition of the Golgi pool of Cdc42 (ref. 46). Interestingly, we determined that the expression of Cdc42 was increased in the GM130-deficient germ cells. Whether the disorganization of the cytoskeleton in the GM130-deficient germ cells is a result of the up-regulation of Cdc42 is not determined in the present study and requires further investigation.

In summary, our study demonstrated inactivation of GM130 causes absence of acrosome, round-shaped sperm head, and aberrant assembly of mitochondrial sheath in mouse model reminiscent of human globozoospermia. The findings in this study highlight the significance of GM130 in acrosome formation. The GM130-deficient mouse model may be a unique and valuable model for investigating the etiology of this human disease.

## Materials and Methods

### Animals

All animal experiments were conducted in accordance with protocols approved by the Animal Care and Use Committee at the Institute of Zoology, Chinese Academy of Sciences (CAS). *GM130*^*flox/flox*^ mice were obtained from Dr Shilai Bao's lab (Institute of Genetics and Developmental Biology, Chinese Academy of Sciences, Beijing, China). The genotyping of *GM130*^−/−^ mouse strain was examined by PCR using DNA isolated from tail tips (Supplementary Figure S2A). All mice were maintained in a C57BL/6; 129/SvEv mixed background. *GM130*^+/−^ mice were generated by crossing *GM130*^*+/flox*^ mice with ZP3-Cre transgenic mice. The genotype of *GM130*^+/−^ mouse strain was examined by PCR using DNA isolated from tail tips as shown in Supplementary Figure S2A.

*GM130*^−/flox^*; Stra8-Cre* mice were obtained by crossing *GM130*^+/−^*; Stra8-Cre* males with *GM130*^*flox/flox*^ females. *GM130*^−/flox^*; AMH-Cre* mice were obtained by crossing *GM130*^+/−^*; AMH-Cr*e males with *GM130*^*flox/flox*^ females. Genotyping was performed via PCR using DNA isolated from tail tips. The primers were as follows: GM130 flox allele forward primer, 5′-TTGTTCAACAGTGGAGCCCT-3′ reverse primer, 5′-TGAAGGCATTTCAACAGGCG-3′ and GM130^*−*^ allele forward primer, 5′- GCCTTTCATTCCTAGCATTTGG-3′ reverse primer, 5′- GGGCTCACACCTGCAACCT-3′.

### Tissue collection and histological analysis

The testes of GM130-deficient and control mice were dissected immediately after euthanasia, fixed in 4% paraformaldehyde (PFA) for up to 24 h, stored in 70% ethanol and embedded in paraffin. Sections were cut to a thickness of 5 *μ*m and mounted on glass slides. After deparaffinization, the sections were processed for immunohistochemistry and immunofluorescence analyses.

### Antibodies

Mouse anti-Ap1 (γ-adaptin) (610386), mouse anti-CDC42 (610929), mouse anti-GM130 (610823), and mouse anti-Golgin-84 (611382) antibodies were purchased from BD Transduction Laboratories (Franklin Lakes, NJ, USA). Mouse anti-TGN46 (ab2809), rabbit anti-TGN46 (ab16059), rabbit anti-VAMP2 (ab181869), rabbit anti-GRASP65 (ab30315), rabbit anti-GOPC (ab37036), rabbit anti-GM130 (ab52649) and mouse anti-RAB27A (ab55667) antibodies were purchased from Abcam (Cambridge, UK). Rabbit anti-MEK1/2 (9122), rabbit anti-Phospho-p44/42 MAPK (Erk1/2) (9101), and rabbit anti-p44/42 MAPK (Erk1/2) (4695) antibodies were purchased from Cell Signaling Technology (Beverly, MA, USA). Chicken anti-PICK1 (NBP1-42829SS) antibody (NB110-60519) was purchased from Novus Biological (Littleton, CO, USA). Mouse anti-β-tubulin (AA12.1), mouse anti-actin (224-236-1) antibodies were purchased from DSHB (Iowa City, IA, USA). Rabbit anti-p115 polyclonal antibody (13509-1-AP) was purchased from Proteintech Group (Wuhan, China). Mouse anti-sp56 (55101) antibody was purchased from QED bioscience (San Diego, CA, USA). Rabbit anti-AFAF antibody was created as previously described.^27^ The mitochondrion-specific vital dye MitoTracker Red CMXRos (M7512) from Invitrogen (Frederick, MD, USA) was used to visualize mitochondrial sheaths.

### Epididymal sperm count and sperm motility analysis

The cauda epididymides were dissected from adult mice. Sperm were extruded from the cauda epididymides and incubated for 30 min at 37 °C. The incubated sperm were subsequently diluted 1:500 and transferred to a hemocytometer for counting. Non-fixed sperm were spread on pre-coated slides for morphological observation or immunostaining. For the mouse sperm motility analysis, a CASA system (Version.12 CEROS, Hamilton Thorne Research, Beverly, MA, USA) was used with the following settings: for cell detection: minimal contrast, 50; minimal cell size, 4 pixels; and 60 frames were acquired at a frame rate of 60 Hz. At least 200 tracks were measured for each specimen at 37 °C with a Slide Warmer (#720230, Hamilton Thorne Research).

### Immunofluorescence

Male mice were killed according to the guidelines of the Ethics Committee of the Institute of Zoology, Chinese Academy of Sciences. The tissues were immediately embedded in optimum cutting temperature compound (OCT, Tissue-Tek) and cut into 8 *μ*m sections using a microtome-cryostat (Leica CM1950, Wetzlar, Germany). Frozen sections were fixed with 4% paraformaldehyde (PFA) for 15 min and washed in phosphate-buffered saline (PBS) three times (pH 7.4). After being blocked with 5% bovine serum albumin (BSA, Sigma, St. Louis, MA, USA) for 30 min, the sections were incubated with primary antibody in 1% BSA at 4 °C overnight. After being washed with PBS, the samples were incubated with FITC- or TRITC-conjugated secondary antibody diluted with PBS for 1 h at 37 °C, washed with PBS and stained with 4′,6-diamidino-2-phenylindole (DAPI). The slides were mounted, and images were captured using a LSM 780/710 microscope (Zeiss, Jena, Germany). For single sperm immunofluorescence, the spermatozoa were washed with PBS three times, spread on 3-aminopropyl-triethoxysilane-coated slides, fixed and stained as previously described.

### Immunohistochemistry

After deparaffinization and rehydration, the paraffin embedded sections were rinsed with PBS (pH 7.4) three times. The sections were subsequently boiled for 15 min in sodium citrate buffer for antigen retrieval. The endogenous peroxidase activity was inhibited by treatment with 3% H_2_O_2_. After blocking with 5% BSA, the sections were incubated with the primary antibody at 4 °C overnight, and this was followed by staining with the HRP-conjugated secondary antibody. Negative controls were prepared without the primary antibody. Finally, the sections were stained with 3, 3'-diaminobenzidine (DAB), and the nuclei were stained with hematoxylin. Images were captured using a Nikon microscope with a CCD (Nikon, Chiyoda-ku, Tokyo, Japan).

### Western blot analysis

Tissue extracts were prepared using a Dounce homogenizer in cold RIPA buffer (25 mM Tris-HCl, pH 7.6, 150 mM NaCl, 1%NP-40, 1% sodium deoxycholate, and 0.1% sodium dodecyl sulfate) supplemented with 1 mM phenylmethylsulfonyl fluoride and a protease inhibitor cocktail (Roche, Indianapolis, IN, USA). The homogenates were centrifuged at 12 000 rpm for 15 min, and the protein concentrations were determined using the Bio-Rad protein assay (Mississauga, ON, Canada). The protein lysates (12 *μ*g) were separated via SDS-PAGE and electro transferred to a nitrocellulose membrane. The membrane was scanned using the ODYSSEY Sa Infrared Imaging System (LI-COR Biosciences, Lincoln, NE, USA).

### TEM

TEM was performed as previously reported.^6^ Briefly, testes and epididymides from adult mice were fixed with 2% glutaraldehyde and 2% paraformaldehyde. The tissues were cut into small pieces, ~1 mm^3^. After fixation with 1% OsO4 in 0.2 M cacodylate buffer, the tissues were dehydrated and embedded in resin. Ultrathin sections were cut with an Ultratome (Leica, Reichert Ultracuts). The sections were stained with uranyl acetate and lead citrate and subsequently examined using a JEM-1400 transmission electron microscope (JEOL, Tokyo, Japan).

### Statistical analysis

All data are presented as the mean±S.E.M. The statistical significance of the differences among the mean values for the different genotypes was analyzed using Student's *t*-tests with a paired two-tailed distribution. The data were considered significant when *P*<0.05 (*) or 0.01(**).
