# Supplementary material for: Globozoospermia and lack of acrosome formation in GM130-deficient mice
Source: Cell Death Dis. 2017 Jan 5;8(1):e2532–. doi: 10.1038/cddis.2016.414 (PMC5386352; doi:10.1038/cddis.2016.414)
Supplement: Supplementary Figure Legends [file cddis2016414x1.docx]

**Supplementary figure legends**

**Fig. S1. The testis weight and body weight of *GM130^-/-^* mice were decreased.** The weights of testes and body of mice at 2 months of age were measured. The testis weights of *GM130^-/-^* mice was reduced approximately 50% compared to control testis (A). The body weight of *GM130^-/-^* mice was also reduced about 50% (B). The ratio of testes weight/body weight was not significantly changed between *GM130^-/-^* and control mice (C). The data are represented as the mean ± S.E.M.

**Fig. S2. Genotyping of *GM130 ^-/-^* mice and the expression of GM130 mRNA in different tissues of control and *GM130^-/-^* mice.** A. The genotype of *GM130^-/-^* mouse strain was examined by PCR using DNA isolated from tail tips. In control mice, 734bp band was amplified. A 734 bp and a 316 bp bands were amplified in *GM130^+/-^* mice. In *GM130^-/-^* mice, only 316 bp bands were detected. B. Expression of GM130 in different tissues of control and *GM130^-/-^* mice was analyzed by Real-time PCR. One of the primers was designed within exon 14. Only the mRNA from wild type allele could be detected by this pair of primers, the mRNA from exon 14-deleted allele will not be detected. The mRNA level of GM130 was dramatically reduced in the testes, lung, liver and kidney of *GM130^-/-^* mice at 2 months of age, indicating that GM130 was completely inactivated in *GM130^-/-^* mice.

**Fig. S3. Spermiogenesis was normal in *GM130^-/flox^; AMH-Cr*e mice.** The histology of the seminiferous tubules was normal in the *GM130^-/flox^; AMH-Cre* testes at 2 months of age (B) compared with the control testes (A). Acrosomes were labeled with anti-AFAF antibody in both the control testes (C, arrowheads) and the testes from the *GM130^-/flox^; AMH-Cre* mice (D, arrowheads). Normal sperm with crescent-shaped heads were observed in the epidydimes of the control mice (E) and the *GM130^-/flox^; AMH-Cre* mice (F). G. single sperm image indicated a normal morphology of the control and *GM130^-/flox^; AMH-Cre* sperm. H. Acrosome-specific protein SP56 was identified in both control and *GM130^-/flox^; AMH-Cre* sperm. I. Normal mitochondrial sheath was observed in control and *GM130^-/flox^; AMH-Cre* sperm.

**Fig. S4. Localization of AP1 and GM130 in control and GM130-deficient germ cells.** The expression of AP1 and GM130 was examined via immunofluorescence. AP1 protein (green) was identified in the germ cells of both the control (D,F) and *GM130^-/-^* testes (A,C), whereas the AP1-positive punctae were fragmented in the GM130-deficient germ cells (A, white arrows) and were smaller than those of the control germ cells (D, white arrows). GM130 protein was only identified in the germ cells of the control testes (E, F red), but not in those of the *GM130^-/-^* testes (B), and the AP1-positive signal was adjacent to GM130 but was not co-localized with GM130 (F, white arrow).

**Fig. S5. Expression of PICK1 was not affected in the testes of *GM130^-/-^* mice.** The expression of PICK1 and GM130 was examined via immunofluorescence. GM130 protein was identified in the germ cells of control testes (E, F green), but not those of the *GM130^-/-^* testes (B, C). PICK1 was identified in the germ cells of both the *GM130^-/-^* (A, C, red) and control (D, F, red) testes and was not co-localized with GM130 in the control testes (D).

**Fig. S6. Disorganized cytoskeleton in germ cells from *GM130^-/-^* testes.** The expression of F-Actin and β-Tubulin was examined via immunofluorescence. A. In the control testes, the actin bundles (Inset, white arrowheads) were symmetrically localized at both sides of the crescent-shaped nucleus of elongated spermatids. B. The actin bundles were disorganized in the GM130-deficient spermatids and identified at only one side of the nucleus (Inset, white arrowheads). C. Microtubules were also symmetrically assembled (Inset, white arrowheads) in the elongated spermatids of the control testes. D. Microtubules were disorganized in the GM130-deficient spermatids (Inset, white arrowheads) and were less condensed.
