# Supplementary figures and images for: Globozoospermia and lack of acrosome formation in GM130-deficient mice
Source: Cell Death Dis. 2017 Jan 5;8(1):e2532–. doi: 10.1038/cddis.2016.414 (PMC5386352; doi:10.1038/cddis.2016.414)

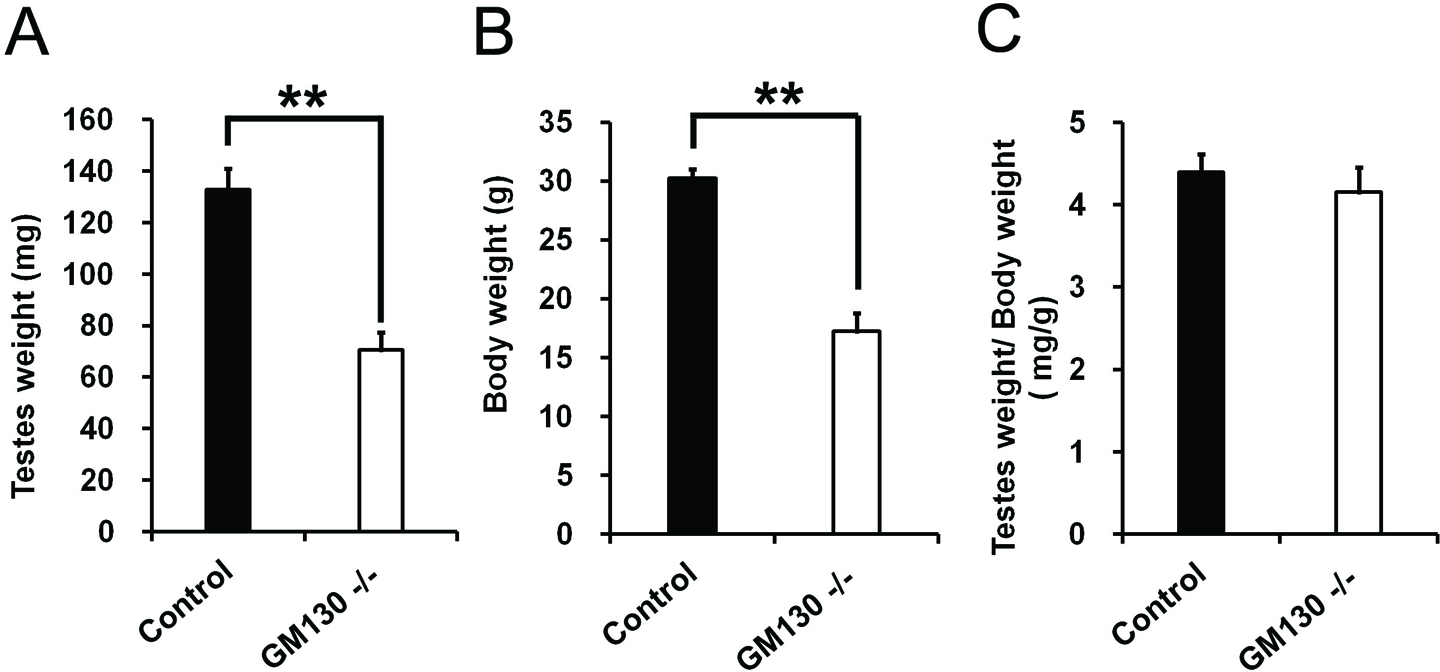

Supplement: Supplementary Figure S1 [file cddis2016414x2.tif]

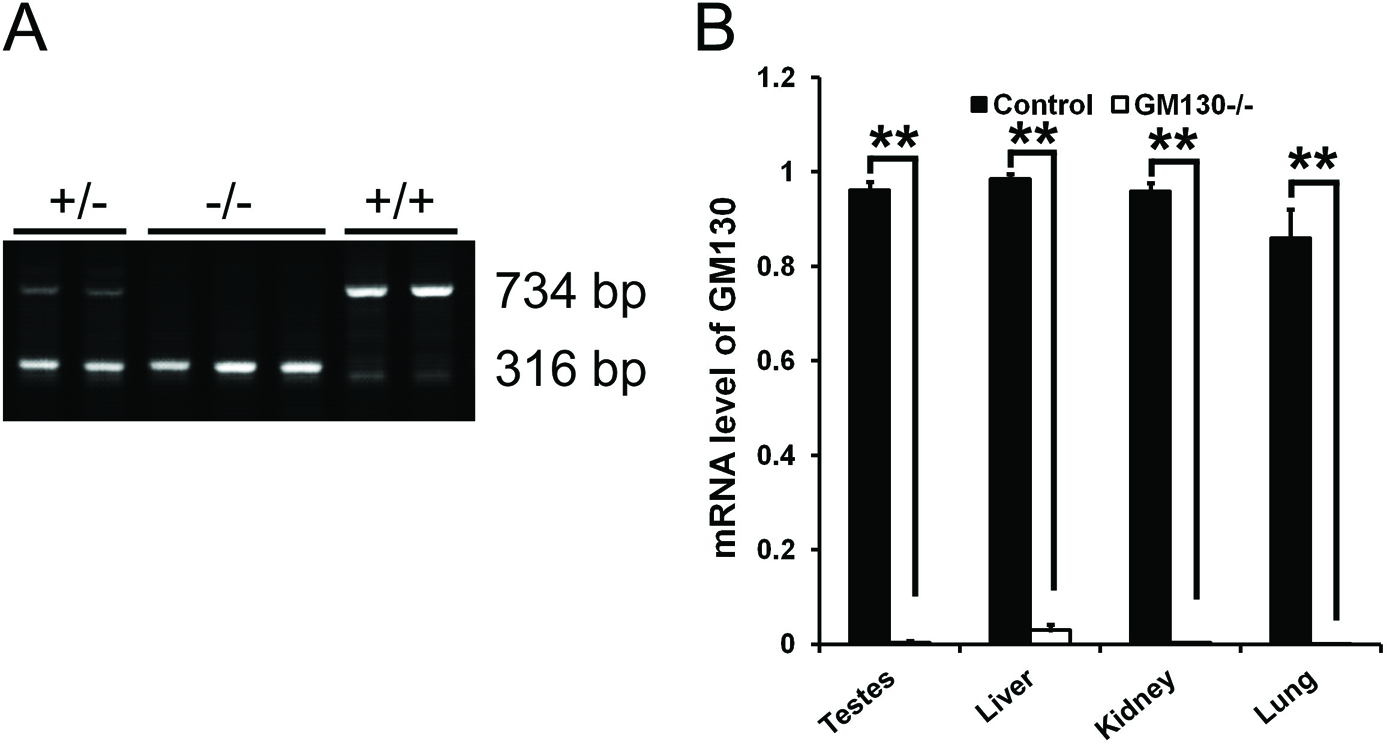

Supplement: Supplementary Figure S2 [file cddis2016414x3.tif]

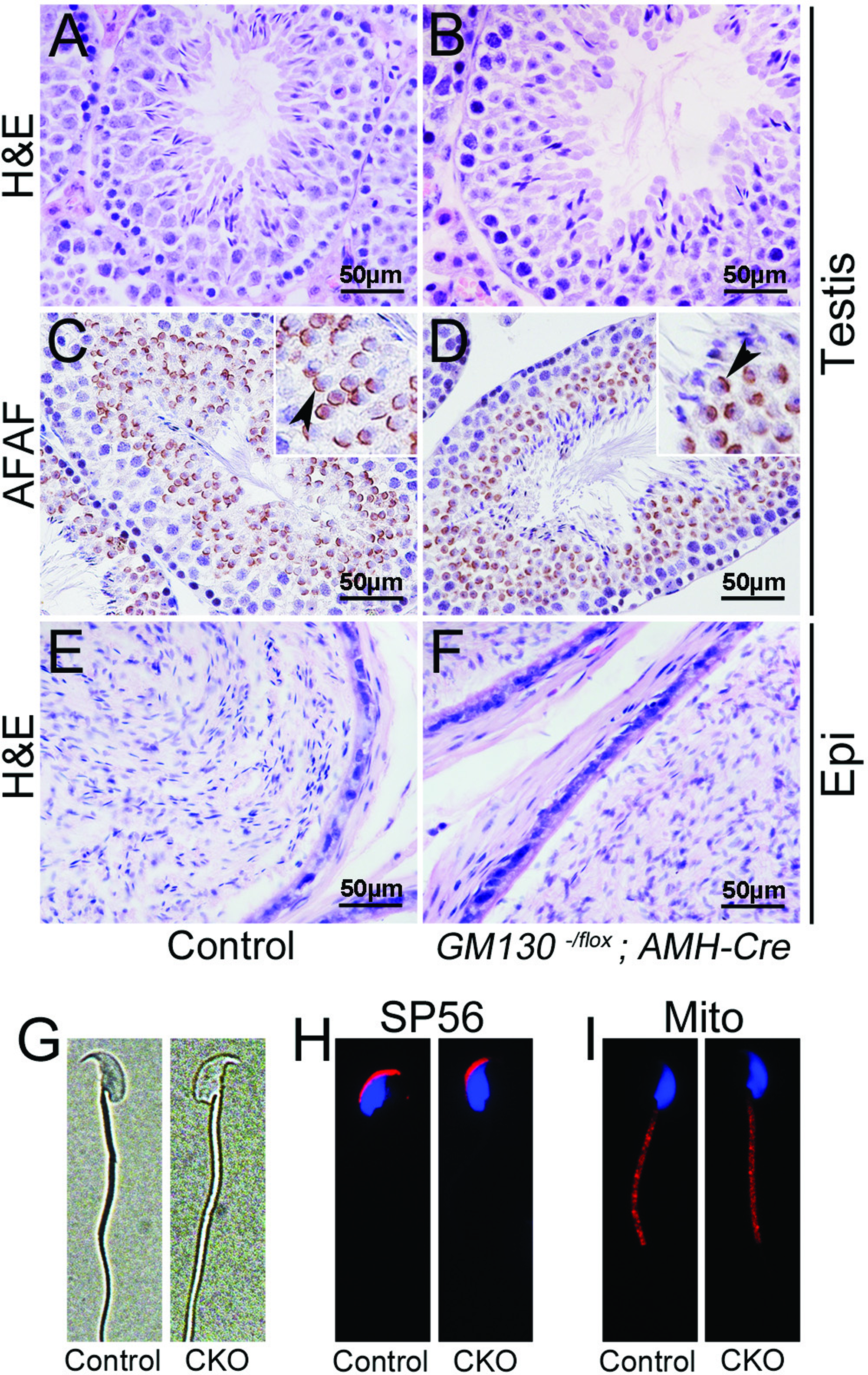

Supplement: Supplementary Figure S3 [file cddis2016414x4.tif]

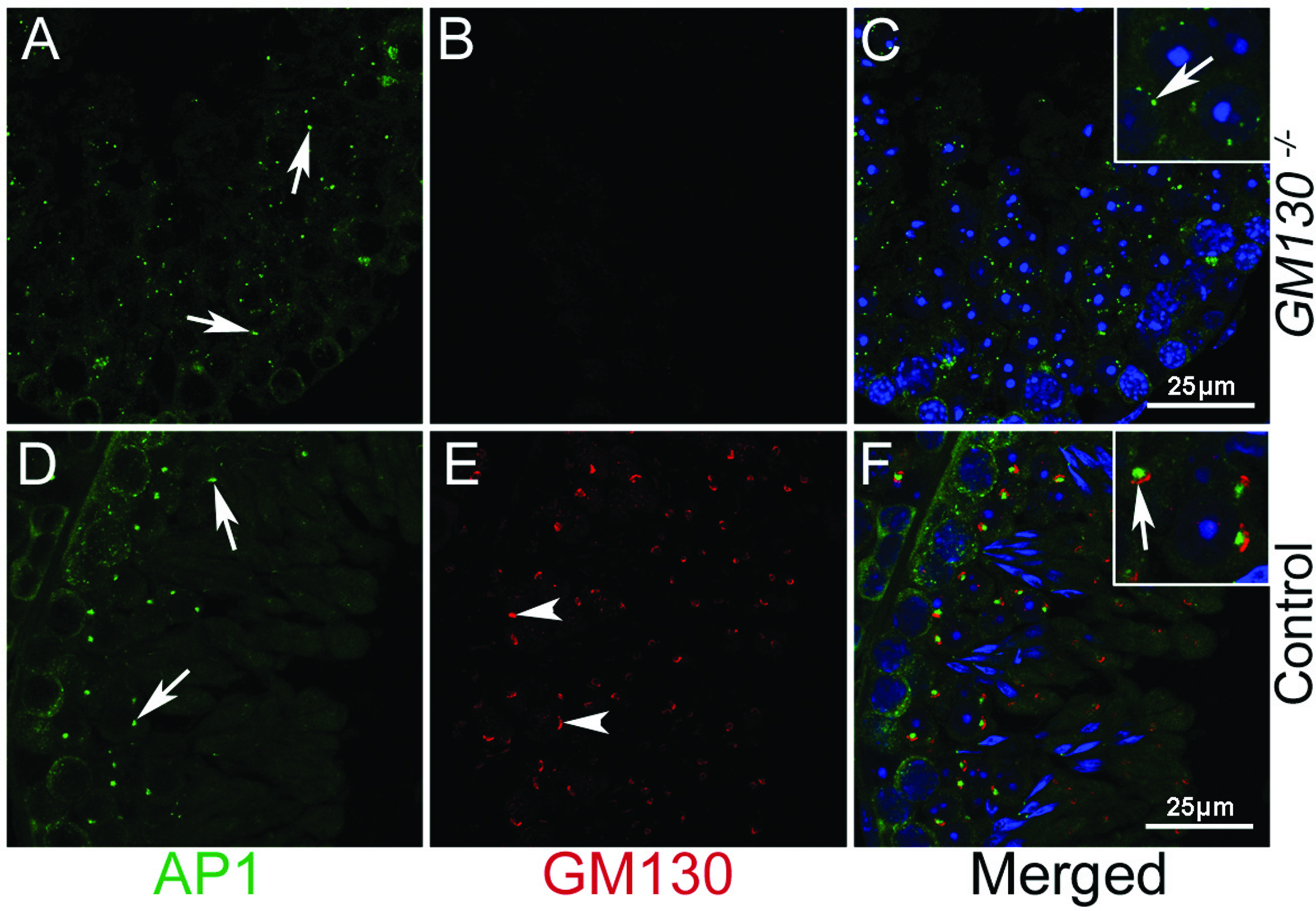

Supplement: Supplementary Figure S4 [file cddis2016414x5.tif]

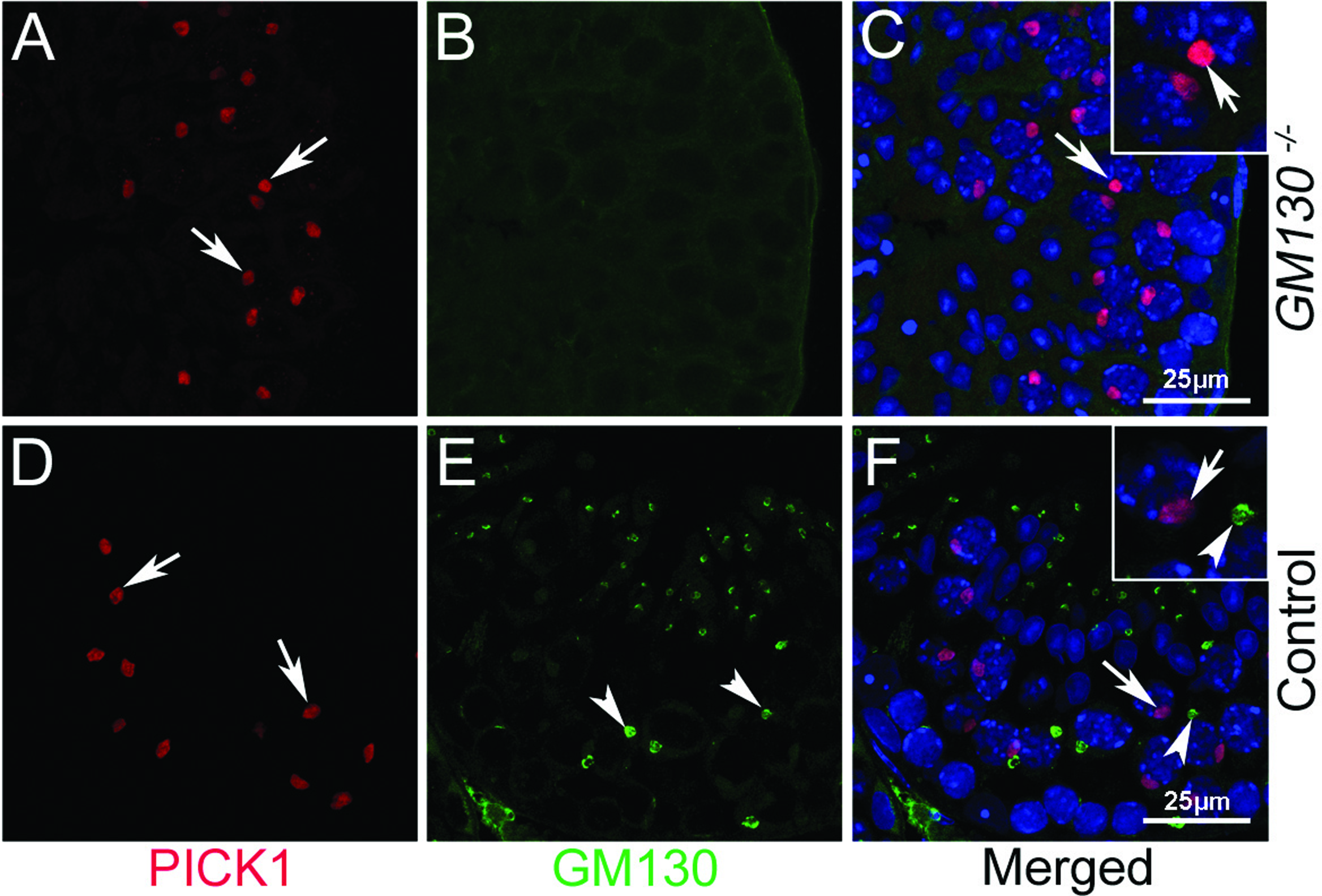

Supplement: Supplementary Figure S5 [file cddis2016414x6.tif]

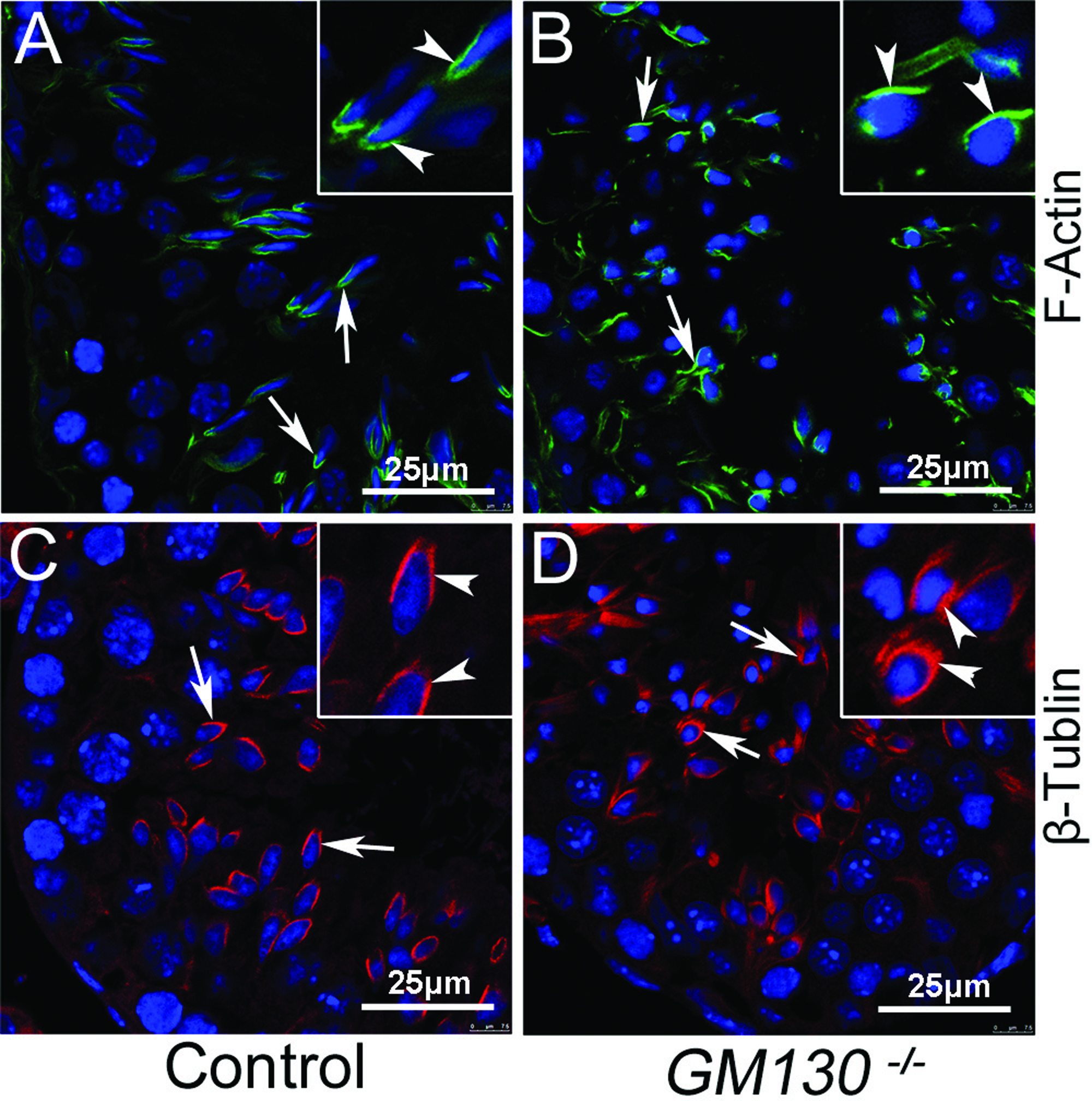

Supplement: Supplementary Figure S6 [file cddis2016414x7.tif]
